# Recurrent postoperative symptomatic epidural hematoma after thoracic decompression surgery: a case report and literature review

**DOI:** 10.3389/fsurg.2026.1882255

**Published:** 2026-10-19

**Authors:** Xuyu Liao, Liujun Zhao, Yong Hu, Liang Yu, Yunlin Chen, Xudong Hu, Weihu Zhang

**Affiliations:** Spine Surgery Center, Ningbo No.6 Hospital, Ningbo, China

**Keywords:** decompression, surgical, hematoma, epidural, spinal, postoperative complications, recurrence, thoracic vertebrae

## Abstract

**Background:**

Postoperative symptomatic epidural hematoma (PSEH) is a rare but potentially devastating complication following thoracic decompression surgery, with an incidence of less than 1%. While isolated PSEH has been well documented, recurrent PSEH,particularly in the thoracic spine which represents an exceptionally rare phenomenon with limited literature available to guide clinical management.

**Case presentation:**

This is a case of a 64-year-old male patient who underwent thoracic decompression surgery for ossification of the ligamentum flavum and developed recurrent PSEH requiring two hematoma evacuations. Due to the timely removing the collagenase covering the dural surface and meticulous intraoperative hemostasis, excellent clinical outcomes were achieved in the end.

**Conclusion:**

Recurrent symptomatic epidural hematoma after thoracic decompression is an exceedingly rare but serious complication. Early recognition, prompt surgical intervention, meticulous management of intraspinal venous hemorrhage and identification of underlying risk factors are essential for optimizing neurological outcomes. This case adds to the limited literature on recurrent PSEH and highlights considerations for intraoperative management.

## Background

Thoracic spine decompression surgery is a common procedure for addressing conditions such as thoracic spinal stenosis caused by ossification of the ligamentum flavum (OLF), ossification of the posterior longitudinal ligament (OPLL), disc protrusion, and traumatic spinal cord compression (1, 2). While generally considered safe, the procedure carries inherent risks, including cerebrospinal fluid leakage, neurological deterioration, and postoperative symptomatic epidural hematoma (PSEH). Although rare, PSEH can be a devastating complication, potentially leading to bowel and bladder dysfunction, saddle anesthesia, motor weakness in the lower extremities, and sexual dysfunction (3). The incidence of PSEH in the thoracic spine is reported to be approximately 1.49% (4), which is notably higher than that following posterior cervical surgery (0.68%), anterior cervical discectomy and fusion (0.44%), and posterior lumbar decompression and fusion (0.10%–0.86%) (5–14).

Conventionally, during thoracic decompression surgery, careful dural handling is required to prevent dural laceration and minimise extensive disruption of the epidural venous plexus. Should bleeding occur, meticulous haemostasis is performed with bipolar electrocoagulation of the fragile, rich epidural venous plexus, combined with packing of the lateral wall of the thoracic canal using gelatin sponge. Bone bleeding is controlled with bone wax. Two negative pressure drainage tubes are routinely placed after the operation to drain the accumulated blood in the wound and prevent postoperative hematoma.

Recurrent PSEH defined as hematoma reformation at the same surgical site after initial evacuation,remains an exceptionally rare event in the literature (15, 16).Given the paucity of published data, guidance for managing such cases is limited. In this report, we describe a case of recurrent symptomatic epidural hematoma following thoracic decompression surgery in a patient with no history of hemorrhagic disease or antiplatelet medication use.

### Case presentation

A 64-year-old male presented on February 1, 2025, with a two-year history of progressive numbness and weakness in both lower extremities. His symptoms had worsened over the past month, rendering him unable to walk and wheelchair-dependent. His medical history was significant for hypertension and hyperlipidemia, managed with valsartan hydrochlorothiazide and atorvastatin. Notably, he had undergone an L4/5 laminectomy at a local hospital 25 years prior. His BMI was 28.4 kg/m².

Physical examination revealed atrophy of the right calf muscle, accompanied by foot drop and clubfoot. Deep tendon reflexes were active in the left patella and hyperactive in the right. The left Babinski sign was positive, while the Hoffmann sign was negative bilaterally. A detailed summary of lower extremity muscle strength is provided in Table 1.

**Table 1:** Muscle strength of lower extremity at admission.

Key Muscles	Muscle Strength	
	Left	Right
iliopsoas	III	III
quadriceps femoris	III	III
hamstring muscles	III	III
tibialis anterior muscle	III	0
extensor digitorum longus	III	0
fibular longus and brevis muscles	III	0
triceps surae	IV	IV

Preoperative CT and MRI demonstrated spinal canal stenosis in the thoracic and lumbar regions, with the most severe compression at the T10-T11 level due to OLF (Figure 1). The patient underwent en-bloc technique for complete resection of the posterior wall of the spinal canal from T10 to L1 that includes the ossified ligamentum flavum by ultrasonic bone scalpel (Figure 2). A medical collagen membrane (GTR Fujian) was placed over the spinal dura mater to prevent adhesions.

**Figure 1:**
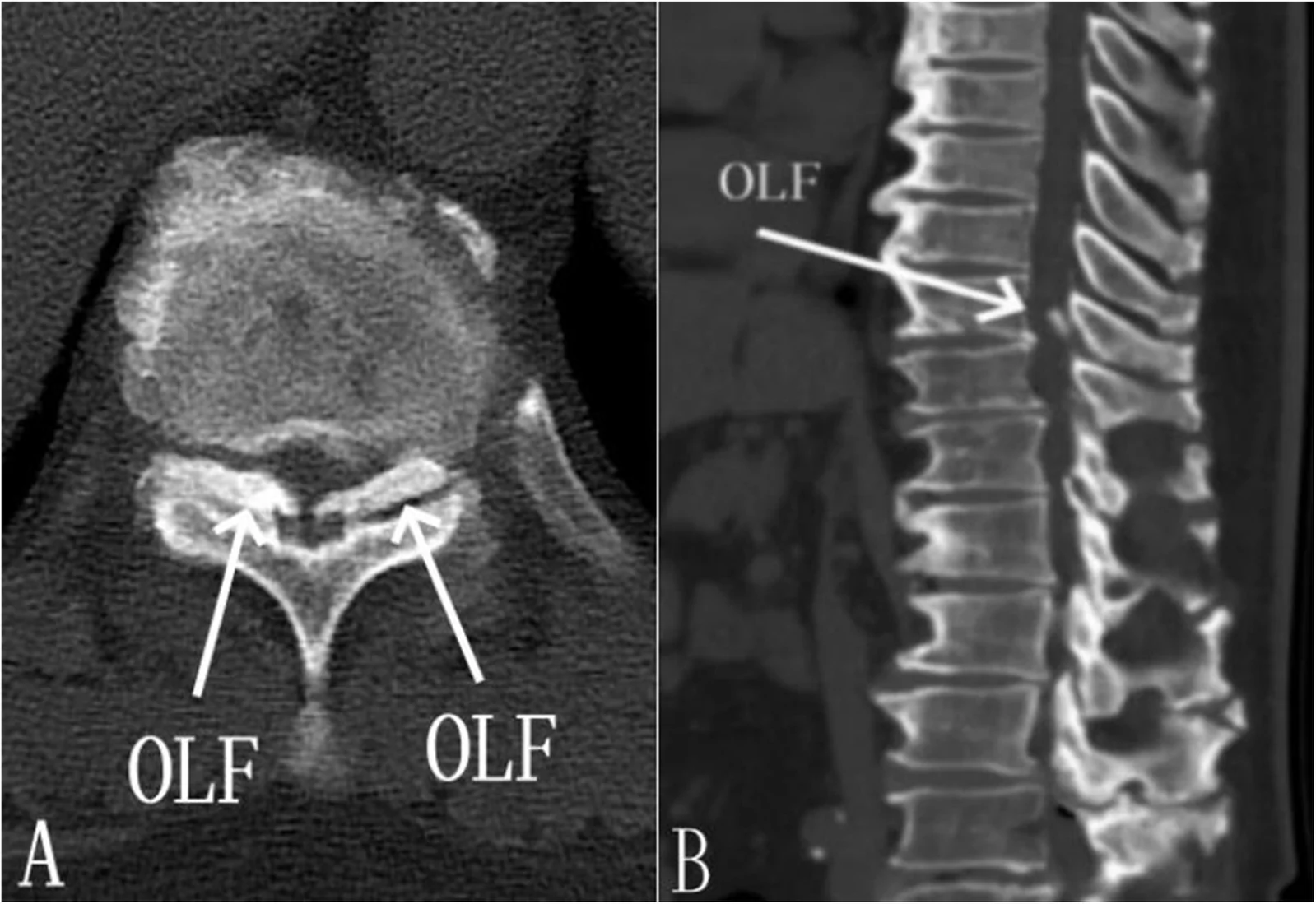
CT revealed that the most severe OLF (ossification of the ligamentum flavum) was located in the T10∼T11 level. **(A)**: Axial CT image demonstrates spinal canal stenosis secondary to OLF (white arrow). **(B)**: Sagittal reformatted-CT image illustrates spinal canal compression by OLF (white arrow).

**Figure 2:**
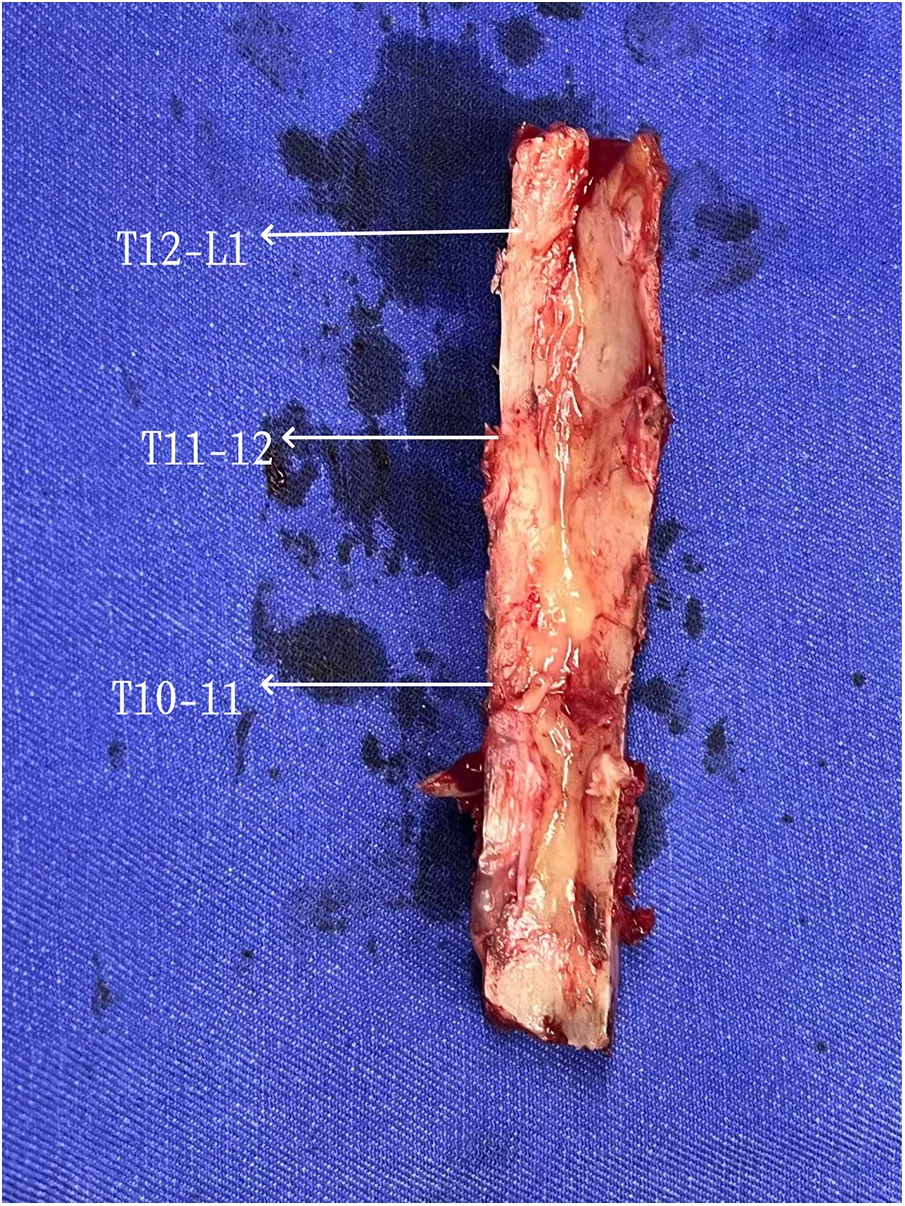
The posterior wall of the spinal canal from T10 to L1 that includes the ossified ligamentum flavum was *en bloc* resected.

Six hours postoperatively, the patient’s iliopsoas and quadriceps muscle strength had recovered to grade IV. However, 13 h after the initial surgery, he reported new-onset numbness in both lower extremities. Muscle strength in the bilateral iliopsoas and quadriceps had deteriorated to grade I, and the left tibialis anterior, fibularis longus, and extensor hallucis longus muscles were also grade I. A diagnosis of PSEH was made. After obtaining informed consent, an emergent hematoma evacuation was performed. Intraoperatively, the collagen membrane was removed, revealing a small amount of clotted blood on the dural surface. Despite irrigation with iced saline, persistent oozing was noted from the venous plexus on the lateral wall of the spinal canal. Four negative-pressure drains were subsequently placed. Six hours after this second surgery, the strength in his bilateral iliopsoas and quadriceps had again improved to grade IV. Tragically, 10 h later, his condition worsened once more, with the same muscles weakening to grade I. An emergent MRI confirmed a recurrent epidural hematoma at the surgical site (Figure 3), necessitating a third operation for evacuation.

**Figure 3:**
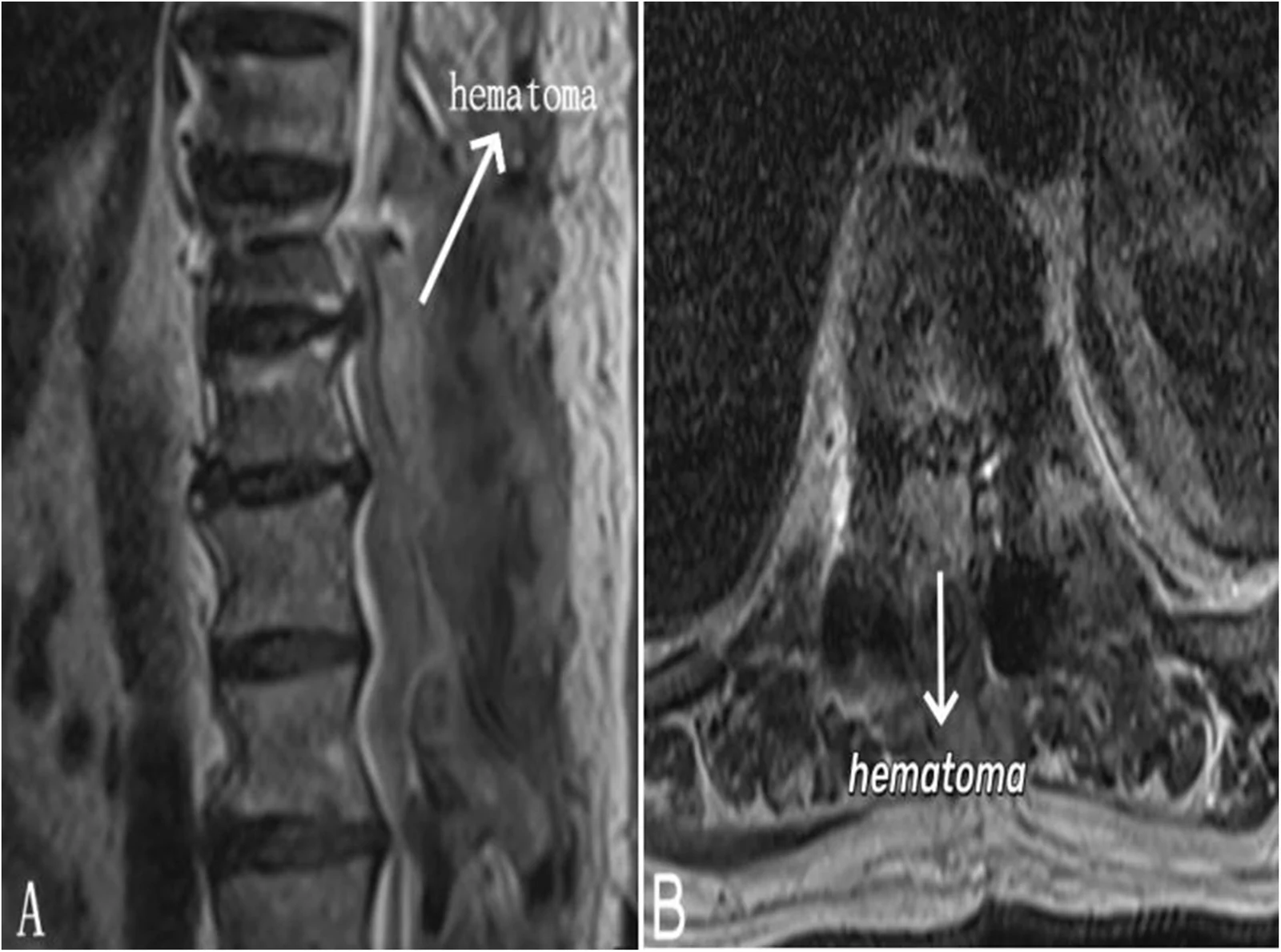
MRI shows an epidural hematoma at the surgical site with compression of the dural sac (white arrow). **(A)**: The sagittal MRI image shows that the dural sac is compressed and flattened. **(B)**: The axial MRI image shows that the dural sac is compressed and flattened.

During this third procedure, the incision was found to be filled with blood clots (Figure 4). Following clot removal, meticulous hemostasis was achieved using bipolar electrocoagulation. A fluid gelatin was applied to fill the lateral wall of the spinal canal, and four large-diameter drainage tubes were placed to prevent further accumulation. Twenty-four hours after this final surgery, the patient’s bilateral iliopsoas and quadriceps strength had recovered to grade III.

**Figure 4:**
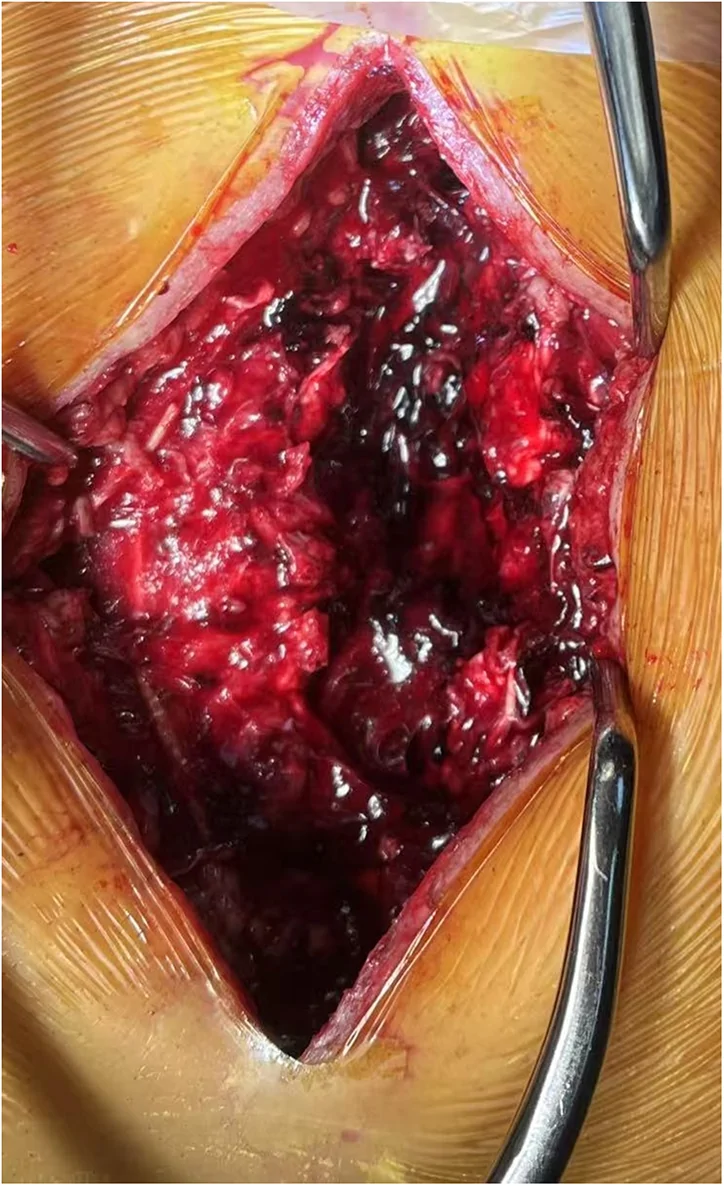
The surgical incision was filled with blood clots.

He was discharged on February 21, 2025, at which point his right foot drop had partially improved, and he was able to walk for 10 min with a walking aid (see Table 2 for a full muscle strength assessment). At a one-month follow-up, his strength had improved further, allowing him to walk 30 steps without crutches. Fourteen months post-discharge, he was able to walk freely and independently for 20 min.

**Table 2:** Muscle strength of lower extrimity at discharge.

Key Muscles	Muscle Strength	
	Left	Right
iliopsoas	IV	IV
quadriceps femoris	IV	IV
hamstring muscles	IV	IV
tibialis anterior muscle	IV	III
extensor digitorum longus	IV	II
fibular longus and brevis muscles	IV	0
triceps surae	IV	IV

## Discussion

The present case demonstrates that PSEH can occur repeatedly, presenting as a serious complication that causes neurologic impairment, including lower extremity paralysis, and saddle anesthesia. Bowel and bladder dysfunction may also result, though this is less commonly observed in the thoracic spine (17).

Current research suggests that the physiological kyphosis of the thoracic spine narrows the space between the paravertebral muscles and the spinal cord when a small hematoma is present. This anatomical compression is believed to explain why PSEH occurs more frequently in the thoracic region than in the cervical or lumbar spine (18, 19). Another contributing factor may be regional differences in spinal cord blood flow. Marcus et al. reported that average blood flow in the cervical and lumbosacral segments is approximately 40% higher than in the thoracic spinal cord (20), potentially making the thoracic segment more vulnerable to ischemic injury following compression.

Anatomically, the epidural venous plexus is an unvalved vascular network with thin and fragile walls that can easily dilate under compression (21, 22). A high occupancy ratio due to thoracic spinal stenosis reduces the elasticity of the epidural venous plexus and impedes blood flow, predisposing it to bleeding. Once hemorrhage occurs, spontaneous hemostasis is difficult to achieve. Consequently, dilatation of the epidural venous plexus contributes to the development of epidural hematoma (23, 24). Therefore, for patients with a high occupancy rate, meticulous surgical technique and rigorous control of venous plexus bleeding are of paramount importance.

Recurrent PSEH defined as hematoma reformation at the same surgical site following initial evacuation that is exceptionally rare. A PubMed review of the English-language literature using the terms “spinal epidural hematoma” and “recurrent” or “relapsed” yielded only seven reported articles documenting recurrent PSEH (Table 3). Given its rarity and potential to cause severe neurological sequelae, recurrent PSEH warrants aggressive treatment (31).

**Table 3:** Patients with recurrence spinal epidural hematoma.

Reference N	Author/year	Age(y)/sex	Procedure	Location	IT to the 1st OP/procedure	IT to the 2st OP/procedure	IT to the 3rd OP/procedure	IT to the 4th OP/procedure	IT to the 5th OP/procedure	Symptom	Primary cause	Outcome
1	Liao/2009 (25)	8/F	evacuation of SSEH	C3–T8	6 years/evacuation of C2-C4					incomplete tetraplegia (ASIA Grade C)	vascular malformation	ICR
		41/M	evacuation of SSEH	L1-5	48 h/ESD					bach pain and weakness in both legs	Child Class C cirrhosis and preoperative coagulopathy	ICR
		53/M	evacuation of SSEH	T4–L3	15 h/ESD					complete paraplegia	ESRD	ICR
2	Lim/2011 (26)	57/M	hemilaminectomy and hematoma evacuation	T11-L3	1 day/total laminectomy(T11-L4) and ESD					low back pain and weakness of the left lower limb	clopidogrel and aspirin	ICR
3	Hernandez D/1982 (27)	67/M	anterior corpectomy and fusion	C6-7	20 days/posterior decompressive laminectomy	17 days/conservative				acute motor deficit	Dual antiplatelet therapy	ICR
4	Anno/2015 (28)	71/M	posterior decompression	C4-5	6 h/ ESD	2 h/ ESD				tetraplegia	Protein S Deficiency	CR
5	Luo/2016 (16)	34/M	posterior laminectomy and hematoma evacuation	L3-4	8 days/NPWT					leg numbness and weakness	hemophilia A	CR
6	Kenneth C/2017 (29)	72/F	posterior decompression	L2-4	2 days/ ESD	1 h/NPDT				back pain and leg pain and leg paraplegia	Dual antiplatelet therapy	ICR
		72/F	posterior fusion	C2-T1	8 days/ ESD	2 days/NPDT				weakness and sensory deficit in the bilateral upper extremities	a baseline coagulopathy secondary to cirrhosis	ICR
		81/M	posterior fusion	C3-4	17 days/ ESD	anesthesia recovery/NPDT				severe neck pain and tetraplegia	pulmonary embolism and deep venous thrombosis	ICR
7	Mohammed M/2019 (30)	24/M	posterior decompression	L3-4	3 days/ evacuation	2 days/ evacuation	28 days/ evacuation	1 day/ evacuation	1day/ evacuation	weakness in both legs and incontinence	Glanzmann thrombasthenia	CR

M, male; F, female; OP, operation; IT, interval time; ESD, evacuation and suction drain; NPDT, negative-pressure dressing therapy; NPWT, negative-pressure wound therapy; CR, complete recovery; ICR, incomplete recovery; ESRD, end-stage renal disease; SSEH, spontaneous spinal epidural hematoma.

Based on our experience with the present case, we believe the following points merit attention when managing PSEH:

Dural Covering Materials as High Risk Factors: Covering substances placed on the dura mater, such as gelatin sponges or collagen membranes, may increase the risk of epidural hematoma formation. These materials can become saturated with blood and surrounded by clot, potentially exerting a mass effect for up to three days postoperatively (32).

Hemostasis of the Intraspinal Venous Plexus: Hemorrhage from the intraspinal venous plexus in the thoracic canal must be actively controlled using bipolar electrocoagulation. If necessary, lateral extension of the decompression may improve exposure of the venous plexus on the ventral aspect of the dura. When bipolar electrocoagulation alone is insufficient, application of liquid gelatin hemostatic agent can be beneficial. In our case, during the second hematoma evacuation, hemostasis was achieved through bipolar electrocoagulation followed by uniform application of liquid gelatin to the expanded lateral wall of the thoracic spinal canal, which yielded excellent hemostatic effect.

Hemostasis Versus Drainage: Effective hemostasis is far more critical than multiple drains. During the first evacuation in this case, failure to achieve adequate hemostasis resulted in hematoma recurrence despite placement of four negative-pressure drains—when the rate of bleeding exceeds the rate of drainage, hematoma re-accumulation is inevitable.

Role of Coagulation Support: Postoperative administration of small, repeated doses of fresh frozen plasma may improve coagulation function and facilitate hemostasis at the surgical site, particularly in patients who have undergone multiple procedures.

In the present case, the collagen membrane contributed to the initial PSEH, whereas hemorrhage from the intraspinal venous plexus was the primary driver of recurrence. Surgeons should remain vigilant to the possibility of PSEH recurrence, particularly in patients undergoing posterior thoracic surgery, and should recognize that dural covering materials, high BMI, hypertension, and hyperlipidemia are potential risk factors for this complication.

## Conclusion

Recurrent symptomatic epidural hematoma is an extremely rare yet severe complication following thoracic decompression. Optimized clinical outcomes rely on early recognition, prompt surgical intervention, meticulous hemostasis of intraspinal venous bleeding, aggressive postoperative supportive care, and identification of underlying risk factors.

## Data Availability

The original contributions presented in the study are included in the article/Supplementary Material, further inquiries can be directed to the corresponding author.

## References

[1] Maruyama J Furuya T Maki S Inoue T Yunde A Miura M. Posterior decompression and fixation for thoracic spine ossification: a 10-year follow-up study. J Clin Med. (2023) 12(17):5701. 10.3390/jcm12175701 37685772 PMC10488937

[2] Ito S Nakashima H Segi N Ouchida J Oishi R Yamauchi I. Ten-year follow-up of posterior decompression and fusion surgery for thoracic ossification of the posterior longitudinal ligament. J Bone Joint Surg Am. (2024) 106(17):1600–9. 10.2106/JBJS.23.01475 39102467

[3] Knusel K Du JY Ren B Kim CY Ahn UM Ahn NU. Symptomatic epidural hematoma after elective posterior lumbar decompression: incidence, timing, risk factors, and associated complications. HSS J. (2020) 16(Suppl 2):230–7. 10.1007/s11420-019-09690-2 33380952 PMC7749891

[4] Wang L Wang H Sun Z Chen Z Sun C Li W. Incidence and risk factors for symptomatic spinal epidural hematoma following posterior thoracic spinal surgery in a single institute. Global Spine J. (2022) 12(6):1175–83. 10.1177/2192568220979141 33334185 PMC9210222

[5] Pivazyan G Kim AJ Aguilera CJ Khan Z Bryant JP Stewart JJ. Incidence, risk factors, and functional outcomes of symptomatic postoperative spinal epidural hematoma: a case-control study. World Neurosurg. (2025) 193:851–60. 10.1016/j.wneu.2024.10.070 39455001

[6] Aikeremu A Liu G. Risk factors of postoperative spinal epidural hematoma after transforaminal lumbar interbody fusion surgery. Neurochirurgie. (2021) 67(5):439–44. 10.1016/j.neuchi.2021.04.012 33915150

[7] Butler AJ Donnally CJ 3rd Goz V Basques BA Vaccaro AR Schroeder GD. Symptomatic postoperative epidural hematoma in the lumbar spine. Clin Spine Surg. (2022) 35(9):354–62. 10.1097/BSD.0000000000001278 34923504

[8] Goldstein CL Bains I Hurlbert RJ. Symptomatic spinal epidural hematoma after posterior cervical surgery: incidence and risk factors. Spine J. (2015) 15:1179–87. 10.1016/j.spinee.2013.11.043 24316117

[9] Djurasovic M Campion C Dimar JR 2nd Glassman SD Gum JL. Postoperative epidural hematoma. Orthop Clin North Am. (2022) 53(1):113–21. 10.1016/j.ocl.2021.08.006 34799017

[10] Tian Y Han Y Li J Wang M Qu Y Fang J. Incidence and risk factors of postoperative epidural hematoma following anterior cervical spine surgery. Beijing Da Xue Xue Bao Yi Xue Ban. (2024) 56(6):1058–64. 10.19723/j.issn.1671-167X.2024.06.018 39690770 PMC11652987

[11] Hohenberger C Zeman F Höhne J Ullrich O-W Brawanski A Schebesch K-M. Symptomatic postoperative spinal epidural hematoma after spinal decompression surgery: prevalence, risk factors, and functional outcome. J NeurolSurg A Cent Eur Neurosurg. (2020) 81:290–6. 10.1055/s-0039-1697024 31935784

[12] Kao F-C Tsai T-T Chen L-H Lai P-L Fu T-S Niu C-C. Symptomatic epidural hematoma after lumbar decompression surgery. Eur Spine J. (2015) 24:348–57. 10.1007/s00586-014-3297-8 24760464

[13] Yin G Ni B. Acute postoperative cervical spinal epidural hematoma. Acta Orthop Traumatol Turc. (2014) 48:437–42. 10.3944/AOTT.2014.13.0133 25230268

[14] Liao Y Tian Y Ye R Tang C Tang Q Ma F. Risk and treatment of symptomatic epidural hematoma after anterior cervical spine surgery. Medicine (Baltimore). (2020) 99:e18711. 10.1097/MD.0000000000018711 31914081 PMC6959927

[15] Morimoto D Kim K Kubota A Kokubo R Iwamoto N Hattori Y. Recurrent cervical spinal epidural hematoma: case report and literature review. NMC Case Rep J. (2020) 7(4):157–60. 10.2176/nmccrj.cr.2019-0253 33062561 PMC7538460

[16] Luo XB Zhou X Wang Q Cai XJ Luo ZP Ma YZ. The classification of recurrent spinal epidural hematoma: a review of the literature and a comparison with the cases. Eur Spine J. (2016 May) 25(Suppl 1):224–9. 10.1007/s00586-016-4506-4 27002614

[17] Liu J-M Deng H-L Zhou Y Chen X-Y Yang D Duan M-S. Incidence and risk factors for symptomatic spinal epidural haematoma following lumbar spinal surgery. Int Orthop. (2017) 41:2297–302. 10.1007/s00264-017-3619-7 28852822

[18] Amiri AR Fouyas IP Cro S Casey AT. Postoperative spinal epidural hematoma (SEH): incidence, risk factors, onset, and management. Spine J. (2013) 13(2):134–40. 10.1016/j.spinee.2012.10.028 23218510

[19] Aono H Ohwada T Hosono N Tobimatsu H Ariga K Fuji T. Incidence of postoperative symptomatic epidural hematoma in spinal decompression surgery. J Neurosurg Spine. (2011) 15(2):202–5. 10.3171/2011.3.SPINE10716 21529204

[20] Marcus ML Heistad DD Ehrhardt JC Abboud FM. Regulation of total and regional spinal cord blood flow. Circ Res. (1977) 41(1):128–34. 10.1161/01.RES.41.1.128 862136

[21] Morikawa M Sato S Numaguchi Y Mihara F Rothman MI. Spinal epidural venous plexus: its MR enhancement patterns and their clinical significance. Radiat Med. (1996) 14(5):221–7. 8988499

[22] Paksoy Y Gormus N. Epidural venous plexus enlargements presenting with radiculopathy and back pain in patients with inferior vena cava obstruction or occlusion. Spine (Phila Pa 1976). (2004) 29(21):2419–24. 10.1097/01.brs.0000144354.36449.2f 15507805

[23] Kou J Fischgrund J Biddinger A Herkowitz H. Risk factors for spinal epidural hematoma after spinal surgery. Spine (Phila Pa 1976). (2002) 27(15):1670–3. 10.1097/00007632-200208010-00016 12163731

[24] Groen RJ Ponssen H. The spontaneous spinal epidural hematoma A study of the etiology. J Neurol Sci. (1990) 98(2-3):121–38. 10.1016/0022-510X(90)90253-J 2243224

[25] Liao C-C Hsieh P-C Lin T-K Lin C-L Lo Y-L Lee S-C. Surgical treatment of spontaneous spinal epidural hematoma: a 5-year experience. J Neurosurg Spine. (2009) 11(4):480–6. 10.3171/2009.4.SPINE08904 19929346

[26] Lim SH Hong BY Cho YR Kim HS Lee JI Kim HW. Relapsed spontaneous spinal epidural hematoma associated with aspirin and clopidogrel. Neurol Sci. (2011) 32(4):687–9. 10.1007/s10072-011-0508-5 21384274

[27] Hernandez D Vinuela F Feasby TE. Recurrent paraplegia with total recovery from spontaneous spinal epidural hematoma. Ann Neurol. (1982) 11(6):623–4. 10.1002/ana.410110611 7114812

[28] Anno M Yamazaki T Hara N Hayakawa K. Recurrent postoperative spinal epidural hematoma in a patient with protein S deficiency. Case Rep Orthop. (2015) 2015:536592–3. 10.1155/2015/536592 26236521 PMC4508477

[29] Foxx KC Mesfin A Molinari R. Negative-pressure dressing management of recurrent postoperative epidural hematoma and uncontrollable intraoperative hemorrhage in posterior spine surgery: report of 4 cases. J Neurosurg Spine. (2018) 28(2):220–5. 10.3171/2017.5.SPINE17387 29192880

[30] Al Barbarawi MM Allouh MZ Audat ZA Aleshawi AJ Al-Shari OM. Unique case of successful surgical treatment of recurrent spinal epidural hematoma after lumbar disc surgery in a glanzmann thrombasthenia patient. Br J Neurosurg. (2023) 37(4):853–5. 10.1080/02688697.2019.1678736 31621418

[31] Hans P Delleuze PP Born JD Bonhomme V. Epidural hematoma after cervical spine surgery. J Neurosurg Anesthesiol. (2003) 15:282–5. 10.1097/00008506-200307000-00020 12826980

[32] Gao WS Ren B An J Zhou XZ. Symptomatic epidural hematoma caused by absorbable gelatin sponge implantation: a case report. Asian J Surg. (2023) 46(12):6035–6. 10.1016/j.asjsur.2023.09.032 37805324

